# Differential expression profiles of plasma exosomal microRNAs in dilated cardiomyopathy with chronic heart failure

**DOI:** 10.1111/jcmm.17789

**Published:** 2023-05-27

**Authors:** Li Zhang, Ge Zhang, Yongzheng Lu, Jiamin Gao, Zhen Qin, Shuai Xu, Zeyu Wang, Yanyan Xu, Yu Yang, Jinying Zhang, Junnan Tang

**Affiliations:** ^1^ Department of Cardiology The First Affiliated Hospital of Zhengzhou University Zhengzhou China; ^2^ Henan Province Key Laboratory of Cardiac Injury and Repair Zhengzhou China; ^3^ Henan Province Clinical Research Center for Cardiovascular Diseases Zhengzhou China

**Keywords:** computational biology, dilated cardiomyopathy, exosome, heart failure, microRNA

## Abstract

As one of the most prevalent heritable cardiovascular diseases, dilated cardiomyopathy (DCM) induces cardiac insufficiency and dysfunction. Although genetic mutation has been identified one of the causes of DCM, the usage of genetic biomarkers such as RNAs for DCM early diagnosis is still being overlooked. In addition, the alternation of RNAs could reflect the progression of the diseases, as an indicator for the prognosis of patients. Therefore, it is beneficial to develop genetic based diagnostic tool for DCM. RNAs are often unstable within circulatory system, leading to the infeasibility for clinical application. Recently discovered exosomal miRNAs have the stability that is then need for diagnostic purpose. Hence, fully understanding of the exosomal miRNA within DCM patients is vital for clinical translation. In this study, we employed the next generation sequencing based on the plasma exosomal miRNAs to comprehensively characterize the miRNAs expression in plasma exosomes from DCM patients exhibiting chronic heart failure (CHF) compared to healthy individuals. A complex landscape of differential miRNAs and target genes in DCM with CHF patients were identified. More importantly, we discovered that 92 differentially expressed miRNAs in DCM patients undergoing CHF were correlated with several enriched pathways, including oxytocin signalling pathway, circadian entrainment, hippo signalling pathway‐multiple species, ras signalling pathway and morphine addiction. This study reveals the miRNA expression profiles in plasma exosomes in DCM patients with CHF, and further reveal their potential roles in the pathogenesis of it, presenting a new direction for clinical diagnosis and management of DCM patients with CHF.

## 1 INTRODUCTION

Dilated cardiomyopathy (DCM), one of the key contributors to chronic heart failure (CHF), is characterized by the dilatation of left ventricle along with systolic dysfunction. Notably, DCM is often correlated with a raised risk of serious arrhythmia, suggesting the pathological involvement of the cardiac conducting system. As the disease progresses, diastolic dysfunction and impaired right ventricular function will develop, ultimately resulting in HF and premature death. Epidemiological surveys revealed that, in the United States, the prevalence of DCM was 36 cases per 100,000 (i.e. 1:2500). Ten thousand people die from DCM each year and 46,000 are hospitalized as a result.^1^, ^2^, ^3^, ^4^ Additionally, population in Africa and Latin America have a higher prevalence compared with United States.^5^ Alarmingly, the prevalence may be even underestimated due to the large number of asymptomatic patients.

DCM's pathogenesis has not been well understood. Familial and genetic predisposition are commonly regard as contributors. Previous studies indicate that several genes may contribute to the initiation, progression and pathology of DCM, including titin (TTN), beta‐myosin heavy chain (MYH7), Cardiac troponin T(TNNT2), Desmoplakin (DSP), lamin A/C (LMNA), type V voltage‐gated cardiac Na channel gene (SCN5A), RNA‐binding motif protein 20 (RBM20) et al.^6^, ^7^, ^8^, ^9^, ^10^ However, although these genes are apparently associated with DCM, limited are directly contributing to the development of DCM due to variations in genetics. Fortunately, with the development of next generation sequencing, an increasing number of DCM‐associated genes have been identified,^11^, ^12^ which opens up the potential for early diagnosis.

Advances in molecular biology over the past decades have revealed that non‐coding RNA (ncRNA) that was once considered as ‘junk’ RNA plays vital roles in regulating distinct cellular processes. Among the ncRNA, miRNAs have been widely studied and better understood. miRNA, which are single‐stranded, conserved RNAs, comprised of approximately 18 ~ 25 nucleotides. It exists in both the supernatant and extracellular vesicles (EVs),^13^, ^14^ exerting regulatory effects via targeting mRNAs for cleavage or translational suppression.^15^ EVs are highly heterogeneous and can be secreted by almost all cell types. Exosomes are the smallest subgroup of EVs, with a dimension of 40–160 nm.^16^ Previous studies suggest that exosomal miRNAs are more stabilized, compared with in supernatants, which precisely reflect the influence on gene regulation.^17^, ^18^, ^19^ For example, Li et al. founded that between healthy and preeclamptic patients, 7 miRNAs were differentially expressed in plasma exosomes, but only one of these can be detected in whole plasma miRNA.^17^ Despite the progression of exosomal miRNAs for diagnosis, limited studies have looked into their potential for DCM early diagnosis.

Hence, we tested the expression profiling of plasma exosomal miRNAs of DCM with CHF patients. By comparative analysis, we identified the differentially expressed exosomal miRNAs. Target gene prediction and functional enrichment analysis were performed via bioinformatics methods, in order to reveal the underlying pathological changes of this disease. The result from this study may provide novel insights for the diagnosis and therapy of DCM with CHF patients.

## 2 MATERIALS AND METHODS

### 2.1 Participants

Approval of this research was obtained from the institutional review board for the First Affiliated Hospital of Zhengzhou University (2020‐KY‐142). The DCM with CHF patients were recruited from the First Affiliated Hospital of Zhengzhou University. DCM was diagnosed according to the diagnostic criteria proposed by the American Heart Association expert consensus panel and the European Society of Cardiology,^20^ and the New York Heart Association (NYHA) criteria were applied to assess the cardiac function grade.^21^

The inclusion criteria for the DCM with HF patients were as follows: (1) left ventricular ejection fraction (LVEF) is equal or lesser than 45% and had heart function grades ranging from II to IV; (2) patients with a history of congestive HF more than 6 months; and (3) informed consent was signed by all participates. The exclusion criteria were as follows: (1) congenital heart disease; (2) CHF induced by other aetiologies, such as coronary heart disease, hypertension and rheumatological disease; (3) patients were complicated with severe liver, kidney and other important organ dysfunction; (4) the history of malignancy; and (5) other connective tissue diseases. The healthy controls all had no any history of cardiovascular disease.

### 2.2 Plasma sample collection

Participants diagnosed with DCN and HF, as well as healthy controls were recruited for the study. Venous blood samples, collected using Na‐EDTA tubes to prevent coagulation, amounted to a minimum volume of 6 mL per participant. The blood samples were subjected to centrifugation at 2000 × *g* for 15 min at room temperature. Subsequently, the upper plasma fraction, with an ideal volume of 2.5 mL per sample, was promptly frozen at −80°C to maintain the stability and integrity of the plasma biomolecules.

### 2.3 Exosome isolation

Plasma exosomes were extracted using Exoquick reagent (EXOQ5A1; System Biosciences, USA) in accordance with the manufacturer's protocol. Briefly, 250 μL of plasma sample was supplemented with 36 μL of ExoQuick Exosome Precipitation Solution. The mixed solution was set for 30 min at 4°C, followed by centrifugation at 1500 × g for 30 min. After the centrifugation, the supernatant was discarded before additional centrifugation (1500 × g for 5 min). Finally, 100 μL of sterile phosphate‐buffered saline (PBS) was add to resuspend exosome pellet. The solution was stored at −80 °C.

### 2.4 Transmission electron microscopy

The transmission electron microscopy (TEM) was used to characterize the morphology character of isolated exosomes. Briefly, PBS was used to dilute extracted exosomes, before being dripped onto a copper mesh grid coated in holey carbon. The solution was set for 10 min, before the excess solution was blotted away with filter paper. The sample was further soaked in 3% of glutaraldehyde for 5 min, prior to the wash process with 10 times washing using de‐ionized water (2 min each time). Afterwards, 4% uranylacetate solution was dropped on the samples for 10 min, followed by 1% methylcellulose fixation for 5 min. Finally, the sample was air‐dried at room temperature for 30 minters and examined by TEM later.

### 2.5 Flow nanoanalyzer

The concentration and diameter distribution of extracted exosomes were determined by Flow NanoAnalyzer (FL Sciences).

### 2.6 Western blot

The extracted exosomes were lysed with precooled RIPA lysis buffer (Solarbio, China) on ice to isolate protein. Equal volumes of protein samples (20 μg) were electrophoresis on a 12.5% sodium dodecyl sulphate‐polyacrylamide gel electrophoresis (SDS‐PAGE) gel, and then, the bands transferred onto a 0.45 μm polyvinylidene fluoride membranes (Millipore, USA). To block the membrane, 5% skimmed milk was applied at room temperature for 2 h, before the overnight incubation with primary antibodies against CD81 (#ab109201, Abcam, USA), CD63 (#ab134045, Abcam, USA), Calnexin (#ab92573, Abcam, USA) at 4°C, after that, incubated with horseradish peroxidase‐conjugated anti‐rabbit secondary antibodies (#ZB‐2301, Zhongshan Golden bridge Biotechnology, China) for 2 h at room temperature. Finally, Amersham Imager 600 (GE Healthcare Life Sciences, USA) and Image J analysis software were used to inspect and analyse protein bands.

### 2.7 RNA extraction

The total RNA that containing miRNA in plasma exosomes was extracted by exoRNeasy (Qiagen, USA) according to the manufacturer's instructions, and the purity and quantity of RNA were assessed by Bioanalyzer 2100 (Agilent, USA). The concentration of RNA is greater than 20 ng/μL as well as its RIN number >7.0 that can meet subsequent experimentation.

### 2.8 MicroRNA library establishment and next generation sequencing

Small RNA library was constructed by using TruSeq Small RNA Sample Prep Kits (Illumina, USA) in accordance with the manufacturer's protocol. Specifically, 3′ and 5′ adapters were ligated with purified RNAs and then synthesized cDNA by using reverse transcription‐polymerase chain reaction (RT‐PCR). cDNA was further purified through High Sensitivity DNA Chip Kit (Agilent, USA). Amplification was carried out by quantitative polymerase chain reaction (qPCR) to establish a MicroRNA library. The single‐end sequencing on an Illumina Hiseq2500 was performed at the LC‐BIO (Hangzhou, China) according to the vendor's recommended protocol.

The raw data were analysed via using ACGT101‐miR (LC Sciences, USA) to remove adapter dimers, low complexities, junk, repeats (http://www.girinst.org/repbase), common RNA families, including tRNA, rRNA, snRNA and snoRNA (http://rfam.sanger.ac.uk/), and sequence length <18 nucleotide (nt) or >26 nt. Based on miRBase 22.0 (http://www.mirbase.org/), the unique sequences with the length of 18 ~ 26 nt were mapped to miRNA sequences, to recognize confirmed miRNAs and pre‐miRNAs (including novel 3p‐ and 5p‐derived miRNAs). Also, after the procedure, data normalization was applied based on the method described previously.^22^, ^23^, ^24^ The remaining unmapped sequences were further screened against Homo sapiens genomic sequences to identify potential novel miRNAs. RNAfold was used to predict the secondary structure of miRNAs (http://rna.tbi.univie.ac.at/cgi‐bin/RNAWebSuite/RNAfold.cgi), in order to confirm the results of putative miRNAs in Homo sapiens. Raw sequencing data from this study are uploaded to the Gene Expression Omnibus (GEO) database (https://www.ncbi.nlm.nih.gov/geo/).

### 2.9 qRT‐PCR analysis for external validation

qRT‐PCR was performed to determine the expression levels of 10 hub miRNAs (hsa‐miR‐6511b‐3p, hsa‐miR‐6741‐5p, hsa‐miR‐1292‐5p, hsa‐miR‐6877‐5p, hsa‐miR‐423‐5p, hsa‐miR‐3138, hsa‐miR‐1304‐3p, hsa‐miR‐5010‐5p, hsa‐mir‐148a‐3p, hsa‐miR‐1306‐3p) in an external population. Internal reference gene U6 was employed. qRT‐PCR assays were performed in triplicate with the following conditions: (1) 95°C for 30 s, (2) 40 cycles of 95°C for 10 s and 60°C for 30 s, (3) 95°C for 15 s, 60°C for 60 s, 95°C for 15 s. Human abdominal aortic wall tissues were homogenized using TissueLyser II (QIAGEN) according to the manufacture's instruction. Total RNA was isolated using TRIzol® reagent (Cat#15596018, Thermo Fisher Scientific) from homogenized tissues. High‐Capacity cDNA Reverse Transcription Kit (Cat#4368813, Thermo Fisher Scientific) was used to generate cDNA. The relative mRNA levels of target genes were quantified using the iTaq UniverSYBR Green SMX 5000 (Cat#1725125, Bio‐Rad) with an ABI PRISM 7900 Sequence Detector system (Applied Biosystems Co, Foster City, CA) following the manufacturer's instructions. The ΔCT (Ct mRNA‐Ct GAPDH) method was used to calculate mRNA relative expression. The relative quantification values for miRNAs were calculated by the 2 − ΔΔCt method.

### 2.10 MicroRNA targeted genes prediction and function enrichment analysis

TargetScan Human 7.0 database^25^ (www.targetscan.org) and miRDB version 3.3a^26^ (mirdb.org/miRDB/) were used to predict miRNA targets. Overrepresentation gene set analysis (ORA) of miRNA targets was performed by computing overlaps with annotated Hallmark gene sets in The Molecular Signatures Database (MsigDB, http://www.broad.mit.edu/gsea/msigdb/index.jsp). As previous studies,^27^ Gene Ontology (GO) Term Enrichment Analysis (http://www.geneontology.org/) and Kyoto Encyclopedia Genes and Genomes (KEGG) Pathway Analysis (http://www.genome.jp/kegg/) were done to predict the function of these target genes. Finally, Metascape (http://metascape.org) were used for the pathway and process enrichment analyses.

### 2.11 Protein–protein interaction network construction and hub genes identification

Protein–protein interaction (PPI) network analysis was done using The Search Tool for the Retrieval of Interacting Genes (STRING) database.^28^ PPI pairs with a combined score >0.4 (medium confidence score) were applied for the PPI network establishment. Subsequently, the Cytoscape software (http://www.cytoscape.org/) was used to visualize the PPI network. As a commonly‐used plugin, CytoHubba calculates the degree of each node in Cytoscape. Nodes with a higher degree or connectivity indicated the highly interacting proteins or genes in the network. In our study, nodes with a degree >5 were recognized as hub genes in the network.

### 2.12 Establishment of the miRNA‐mRNA regulatory network

The miRNet database (https://www.mirnet.ca) is an easy‐to‐use platform for the visualization and analysis of miRNA‐centric network, designing to help unravel microRNA functions through consolidating existing knowledge with users' data.^29^ The miRNet database was conducted to recognize miRNAs targeting hub genes in this study. The miRNA‐mRNA network of DCM accompanied with CHF was constructed by Cytoscape software.

### 2.13 Calculation of the global difference between a pair of expression profiles

We applied two different methods to calculate the global difference between a pair of expression profiles^30^: The Euclidean distance,

$$\text{RMSD}=\sqrt{\sum_{i=1}^{n}{\left(\log2_{2}x_{i}-\log2_{2}y_{i}\right)}^{2}/n}$$

where *x*_*i*_ and *y*_*i*_ are the expression of hub miRNA i over two expression profiles (DCM‐Exo and Health‐Exo) with *p* and *q* samples (*x* ^1^, *x* ^2^, …, *x* ^*p*^), (*y* ^1^, *y* ^2^, …, *y* ^*q*^).

### 2.14 Dimensional reduction analysis

The uniform manifold approximation and projection (UMAP) using the umap‐learn package (https://umap‐learn.readthedocs.io/en/latest/) was applied for dimensional reduction of internal transcriptomics data based on DE miRNAs. To test the discrimibility of identified hub miRNAs, we performed principal coordinates analysis (PCoA) and analysis of similarities (ANOSIM) on them. Bray–Curtis dissimilarity matrix was calculated by beta_diversity.py, and Bray–Curtis diversity was calculated using the R package Vegan with the function vegdist.

### 2.15 Statistical analysis

All statistical tests were two‐sided. *p*‐value <0.05 and FDR <0.05 were suggested to be statistically significant. The mean ± standard deviation for descriptive statistics was used for continuous variables with a normal distribution. The Wilcoxon rank‐sum test or Student's t‐test was applied to compare continuous variables, and categorical variables were compared through the chi‐squared or Fisher exact test.^31^ All data processing, statistical analysis and plotting were conducted with R 4.1.3 software.

## 3 RESULTS

### 3.1 Identification of isolated plasma exosomes

**FIGURE 1:**
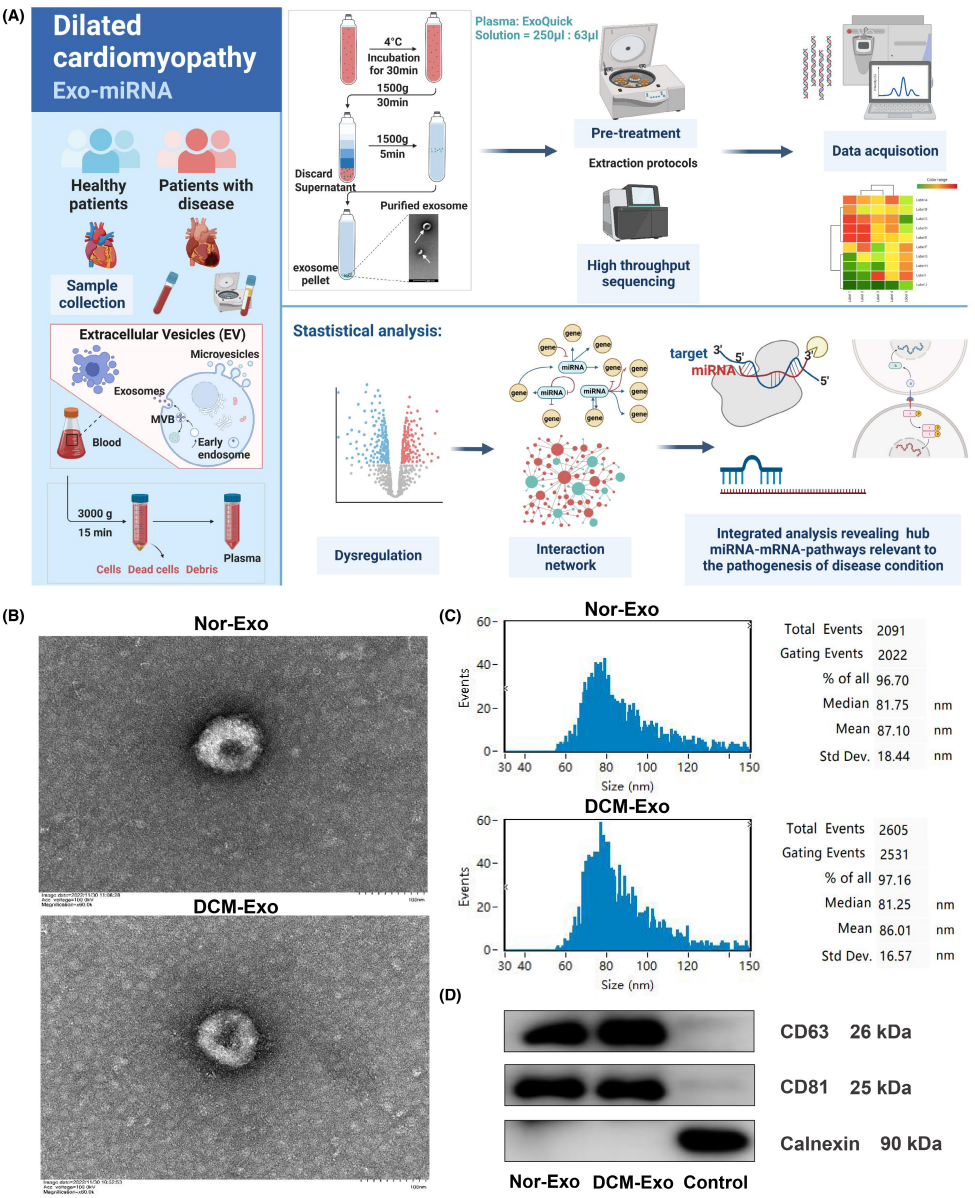
Characterization and internalization of DCM‐Exo and Nor‐Exo. (A) Complete workflow for high‐throughput sequencing and analysis of plasma exosome samples of DCM with CHF. (B) Electron microscopic images of isolated exosomes. Scale bar, 100 nm. (C) Results of high sensitivity flow cytometry for nanoparticle analysis of exosomes. (D) Western blotting revealed CD81, CD63 proteins and EV negative marker protein Calnexin in exosome and cell control samples.

An overall flowchart of the study design is shown in Figure 1A. Plasma exosomes were characterized for their diameter, morphology and the exosome surface proteins such as CD63 and CD81. TEM images (Figure 1B) demonstrated that isolated plasma exosomes were cuplike constructs. High sensitivity flow cytometry for nanoparticle analysis revealed that both DCM‐Exo and Nor‐Exo had a similar median diameter about 81 nm (Figure 1C). Moreover, these exosomes were all positive expression of CD63 and CD81 proteins but negative expression of Calnexin proteins on Western blotting (Figure 1D). As a result, we have accurately identified plasma exosomes for the further high‐throughput sequencing.

### 3.2 Exosomal miRNA profile of DCM with CHF patients by RNA sequencing

**FIGURE 2:**
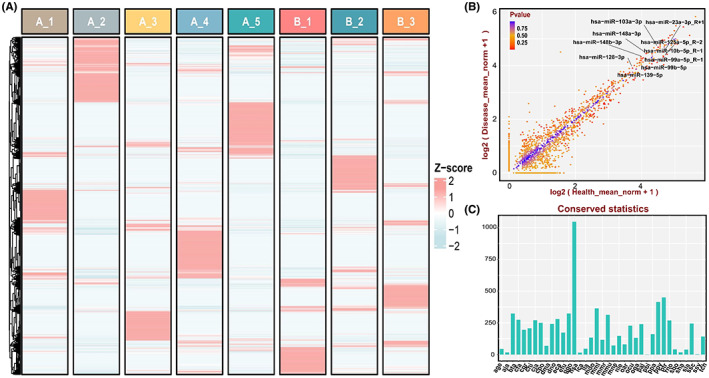
Landscape of exosomal miRNA profile. (A) Heatmap of all expressed exosomal miRNAs between DCM with CHF and controls, where A represented the DCM group and B represented the control group. (B) Scatter plot of all expressed exosomal miRNAs between DCM with CHF and controls. (C) The conservatism analysis of all expressed exosomal miRNAs.

A total of 3687 miRNAs were detected in the plasma exosome in the plasma exosome of the DCM patients with CHF patients and healthy controls (Table S1). The heatmap demonstrated the landscape of miRNA profile (Figure 2A). Scatter plot demonstrated all expressed exosomal miRNAs between DCM with CHF and controls (Figure 2B). The conservation analysis of all expressed exosomal miRNAs also had been performed (Figure 2C).

**FIGURE 3:**
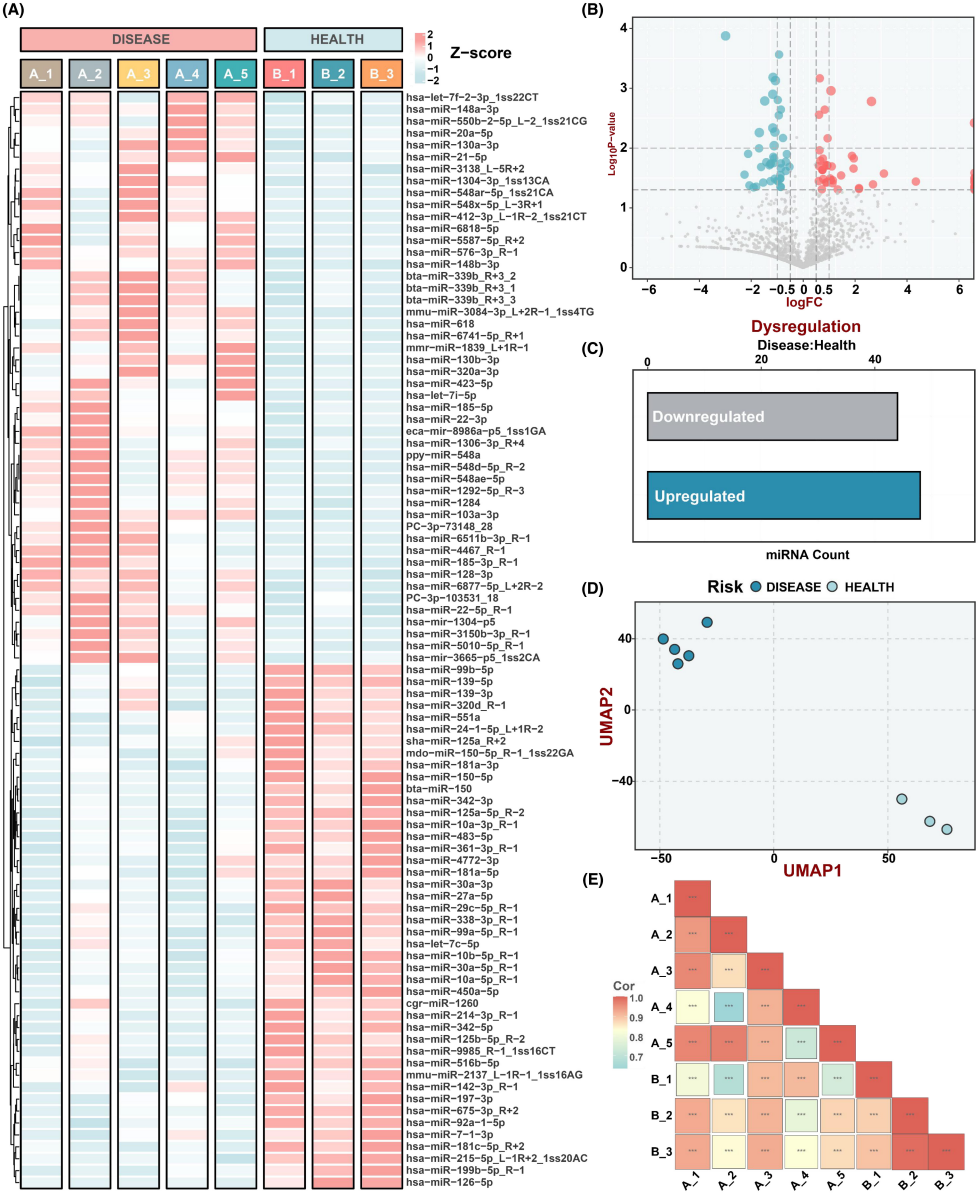
Expression of plasma exosomal microRNAs from DCM with CHF. (A) The heat map shows the 92 DE‐miRNAs expression profile under the threshold absolute logFC >0.05 and *p* < 0.05, where A represented the DCM group and B represented the control group. (B) The volcano plot of DE‐miRNAs. Red dots represent upregulated miRNAs; grey dots not‐significant miRNAs; and blue dots downregulated miRNAs. (C) Numbers of DE‐miRNAs in the two modules. (D) Dimension reduction analysis of DCM and control samples for the DE‐miRNAs expression profile. Dark blue dots represent DCM with CHF samples; light blue dots represent control samples. (E) Pearson correlation analysis plot of normal samples and DCM samples.

A total of 98 miRNAs were expressed significantly differently between the two groups under the threshold of *p* < 0.05. Among them, 50 miRNAs were upregulated and 48 miRNAs were downregulated. Furthermore, 85 miRNAs were borderline significantly different (*p* = 0.05–0.1), including 61 that were upregulated in DCM with HF patients and 24 that were downregulated (Table S1). Based on *p*‐value less than 0.05 and |log fold change (FC)| more than 0.5, we determined 92 DE‐miRNAs for subsequent analysis, including 48 upregulated and 44 downregulated miRNAs (Figure 3A, Table 1). The details of downregulated miRNAs were further demonstrated in the volcano map and bar plot (Figure 3B,C). We further performed Pearson correlation analysis and UMAP analysis on the miRNA profiles to evaluate the differences and similarities in miRNA expression between the samples of DCM with CHF patients and healthy control participants (Figure 3D,E). These results have revealed a significant difference between DCM and healthy individuals on their plasma exosomal miRNA landscapes.

**TABLE 1:** Information of the 92 DE‐miRNAs in DCM with CHF.

Index	Upregulation			Downregulation		
	miR‐name	Log FC	*p*‐value	miR‐name	Log FC	*p*‐value
1	hsa‐miR‐103a‐3p	0.64	0.00068	hsa‐miR‐675‐3p_R+2	−2.99	0.00013
2	hsa‐miR‐148a‐3p	1.07	0.0011	hsa‐miR‐99b‐5p	−0.93	0.00027
3	hsa‐miR‐6877‐5p_L+2R‐2	2.63	0.00166	hsa‐miR‐215‐5p_L‐1R+2_1ss20AC	−1.16	0.00066
4	hsa‐miR‐148b‐3p	0.83	0.00228	hsa‐miR‐125a‐5p_R‐2	−1.07	0.00074
5	hsa‐miR‐128‐3p	0.61	0.00277	hsa‐miR‐338‐3p_R‐1	−1.17	0.00126
6	mmu‐miR‐3084‐3p_L+2R‐1_1ss4TG	Inf	0.0038	hsa‐miR‐29c‐5p_R‐1	−0.98	0.00157
7	hsa‐miR‐130b‐3p	0.94	0.00687	hsa‐miR‐92a‐1‐5p	−1.48	0.00163
8	hsa‐miR‐21‐5p	0.63	0.0109	hsa‐miR‐139‐5p	−0.86	0.00227
9	hsa‐miR‐1304‐3p_1ss13CA	1.88	0.0135	hsa‐miR‐181c‐5p_R+2	−0.94	0.00283
10	eca‐mir‐8986a‐p5_1ss1GA	1.95	0.0149	hsa‐miR‐10a‐3p_R‐1	−1.17	0.00462
11	hsa‐let‐7i‐5p	0.76	0.015	hsa‐miR‐483‐5p	−1.09	0.00539
12	hsa‐miR‐185‐3p_R‐1	0.66	0.0162	hsa‐miR‐516b‐5p	−1.69	0.00551
13	mmr‐miR‐1839_L+1R‐1	0.89	0.0192	hsa‐miR‐99a‐5p_R‐1	−0.78	0.00679
14	bta‐miR‐339b_R+3_1	0.93	0.0195	hsa‐miR‐10b‐5p_R‐1	−1.12	0.00912
15	bta‐miR‐339b_R+3_3	0.93	0.0195	mmu‐miR‐2137_L‐1R‐1_1ss16AG	−1.7	0.0101
16	bta‐miR‐339b_R+3_2	0.93	0.0195	hsa‐miR‐551a	−2.12	0.0125
17	hsa‐miR‐22‐5p_R‐1	0.61	0.02	hsa‐miR‐181a‐3p	−0.63	0.0126
18	hsa‐mir‐1304‐p5	1.1	0.0203	hsa‐miR‐9985_R‐1_1ss16CT	−1.15	0.014
19	hsa‐miR‐3138_L‐5R+2	1.94	0.022	hsa‐miR‐4772‐3p	−1.12	0.0148
20	hsa‐miR‐548d‐5p_R‐2	0.74	0.0232	hsa‐miR‐181a‐5p	−0.67	0.0169
21	hsa‐miR‐548ae‐5p	0.74	0.0232	hsa‐miR‐342‐5p	−1.42	0.0172
22	ppy‐miR‐548a	0.74	0.0232	hsa‐miR‐10a‐5p_R‐1	−1.13	0.0178
23	hsa‐miR‐618	Inf	0.0259	hsa‐miR‐125b‐5p_R‐2	−0.86	0.018
24	hsa‐miR‐6741‐5p_R+1	3.11	0.0267	cgr‐miR‐1260	−1.25	0.0181
25	hsa‐miR‐5010‐5p_R‐1	1.46	0.0287	hsa‐miR‐197‐3p	−1.26	0.0186
26	hsa‐miR‐412‐3p_L‐1R‐2_1ss21CT	Inf	0.0312	hsa‐miR‐30a‐3p	−1.3	0.0194
27	hsa‐miR‐185‐5p	1.04	0.0322	hsa‐let‐7c‐5p	−0.53	0.0206
28	hsa‐let‐7f‐2‐3p_1ss22CT	0.77	0.0328	hsa‐miR‐24‐1‐5p_L+1R‐2	−1.62	0.0207
29	hsa‐miR‐4467_R‐1	Inf	0.0329	hsa‐miR‐139‐3p	−0.86	0.0217
30	hsa‐miR‐1284	1.06	0.0342	hsa‐miR‐7‐1‐3p	−0.67	0.0241
31	hsa‐miR‐1306‐3p_R+4	1.14	0.0346	bta‐miR‐150	−2.26	0.0278
32	hsa‐miR‐20a‐5p	0.6	0.0356	hsa‐miR‐320d_R‐1	−0.88	0.0282
33	hsa‐miR‐5587‐5p_R+2	Inf	0.0357	hsa‐miR‐361‐3p_R‐1	−0.88	0.0322
34	hsa‐miR‐6511b‐3p_R‐1	4.34	0.0363	hsa‐miR‐199b‐5p_R‐1	−1.1	0.0326
35	hsa‐miR‐320a‐3p	0.89	0.0374	sha‐miR‐125a_R+2	−0.89	0.0329
36	hsa‐miR‐22‐3p	0.89	0.0377	hsa‐miR‐142‐3p_R‐1	−1	0.0347
37	hsa‐miR‐3150b‐3p_R‐1	Inf	0.0382	hsa‐miR‐27a‐5p	−0.98	0.0357
38	hsa‐miR‐423‐5p	1.13	0.0386	hsa‐miR‐30a‐5p_R‐1	−1.26	0.0357
39	hsa‐miR‐1292‐5p_R‐3	2.69	0.0404	hsa‐miR‐214‐3p_R‐1	−1.54	0.0377
40	hsa‐miR‐550b‐2‐5p_L‐2_1ss21CG	Inf	0.0433	hsa‐miR‐150‐5p	−2.07	0.042
41	hsa‐miR‐548ar‐5p_1ss21CA	Inf	0.0455	mdo‐miR‐150‐5p_R‐1_1ss22GA	−1.81	0.0441
42	hsa‐miR‐548x‐5p_L‐3R+1	Inf	0.0455	hsa‐miR‐450a‐5p	−0.88	0.0444
43	hsa‐mir‐3665‐p5_1ss2CA	2.15	0.0467	hsa‐miR‐126‐5p	−0.86	0.0446
44	PC‐3p‐73148_28	Inf	0.0479	hsa‐miR‐342‐3p	−1.91	0.0454
45	PC‐3p‐103531_18	2.15	0.0482			
46	hsa‐miR‐130a‐3p	0.72	0.0489			
47	hsa‐miR‐576‐3p_R‐1	1.33	0.0492			
48	hsa‐miR‐6818‐5p	Inf	0.0499			

### 3.3 Analysis of DE‐miRNAs targeted genes

TargetScan and miRanda, two target prediction software, were used to predict the possible targets of the 92 DE‐miRNAs. Specifically, all results are screened based on their scoring criteria: the targets with Targetscan context score percentile less than 80 and miRanda max energy greater than or equal to −80 were excluded. Then, the intersection of the remaining results from two types of software was taken as the final targets of the 92 DE‐miRNAs. Finally, based on the above workflow, a total of 75 of 1176 matched genes were identified as candidate targets (Table S2). These 75 targets genes could play majors roles in the pathogenic regulatory mechanism of DCM.

### 3.4 Overrepresentation gene set analysis of DE‐miRNAs targeted genes

**FIGURE 4:**
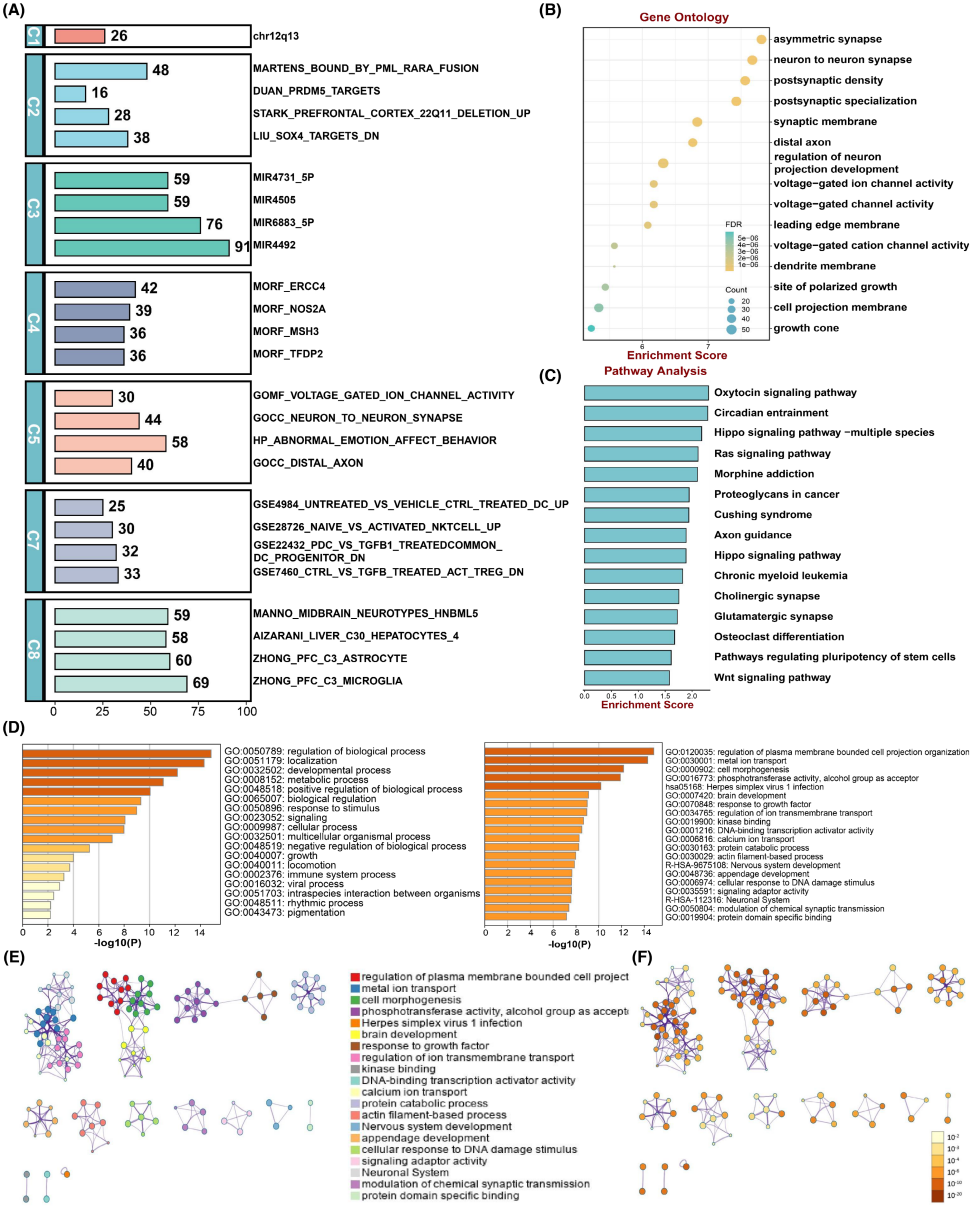
Biological implications of DE‐miRNAs target genes. (A) The MSigDB signature analysis of DE‐miRNAs target genes. C1: positional gene sets; C2: curated gene sets; C3: regulatory target gene sets; C4: computational gene sets; C5: ontology gene sets; C7: immunologic signature; C8: cell type signature gene sets. (B) Bubble plot of GO enrichment analysis of DE‐miRNAs target genes. (C) The top 15 most significant KEGG pathway terms. (D) Top 20 clusters from Metascape pathway and process enrichment analysis of DE‐miRNAs target genes, coloured by *p*‐values. (E,F) Network of enriched terms of DE‐miRNAs target genes. Terms with a similarity >0.3 are linked by edges: (E) coloured by enriched terms, where nodes that share the same enriched term are typically close to each other; (F) coloured by *p*‐value, where terms containing more genes tend to have a more significant *p*‐value.

To confirm the accuracy and reliability of targeted genes prediction, we performed gene set analysis on 75 targets of DE‐miRNAs by using MsigDB. As shown in Figure 4A, consistent with our data sources, these targets significantly enriched in C3 regulatory target gene sets and C8 cell type signature gene sets. This analysis indicates that the predicted target genes belong to gene sets that were closely correlated with the plasma exosomal miRNAs of DCM with CHF.

### 3.5 GO Enrichment analysis of DE‐miRNAs targeted genes

GO software was used to annotate the functions of the 75 predicted targets of the DE‐miRNAs, and GO analysis results have been conventionally grouped into three key categories: (i) biological process, (ii) cellar component and (iii) molecular function. Taken as a whole, the 75 targets of DE‐miRNAs remarkably enriched in asymmetric synapse, neuron to neuron synapse and postsynaptic density and others. (Figure 4B), and these three terms all belong to cellular components. As to biological process, the top 3 significantly enriched terms were the regulation of neuron projection development, the positive regulation of cell projection organization and the regulation of membrane potential. For molecular function, the top 3 significantly enriched terms were voltage‐gated ion channel activity, voltage‐gated channel activity and voltage‐gated cation channel activity (Table S3). These prominently enriched terms may help us to further understand the role which DE‐miRNAs played in the occurrence and progress of DCM with CHF.

### 3.6 Enrichment KEGG analysis of DE‐miRNAs target genes

Different than GO terms enrichment analysis, KEGG enrichment analysis could provide an insight for researcher into quite complicated molecular mechanism consisting in DCM with CHF. A total of 25 markedly enriched pathways were detected (*p*‐value <0.05). In order to explore the vital mechanistic pathways of the targeted genes of DE‐miRNAs, we identified the top 15 statistically remarkably enriched pathways based on KEGG pathway results (Figure 4C). Among these cascades, oxytocin signalling pathway, circadian entrainment and hippo signalling pathway‐multiple species seemed to be intimately associated with exosomal miRNAs in DCM with CHF. Furthermore, previous studies have demonstrated that RAS signalling pathway, axon guidance and glutamatergic synapse also strongly associated with the physiological and pathophysiological processes of DCM.^32^, ^33^, ^34^

### 3.7 Metascape functional enrichment analysis

Next, we preformed Metascape pathway and process enrichment analysis of 1176 predicted targets of DE‐miRNAs integrating the following ontology sources: GO Biological Processes, GO Molecular Functions, KEGG Pathway, Hallmark Gene Sets, Reactome Gene Sets and Canonical Pathways. The most significantly enriched terms were regulation of plasma membrane bounded cell projection organization (GO:0120035), metal ion transport (GO:0030001) and metal ion transport (GO:0000902). The top 20 clusters enrichment terms were showed in Figure 4D. To further obtain the relationships between these top 20 terms, a functional annotation network was constructed (Figure 4E,F).

### 3.8 Protein–protein interaction network analysis

**FIGURE 5:**
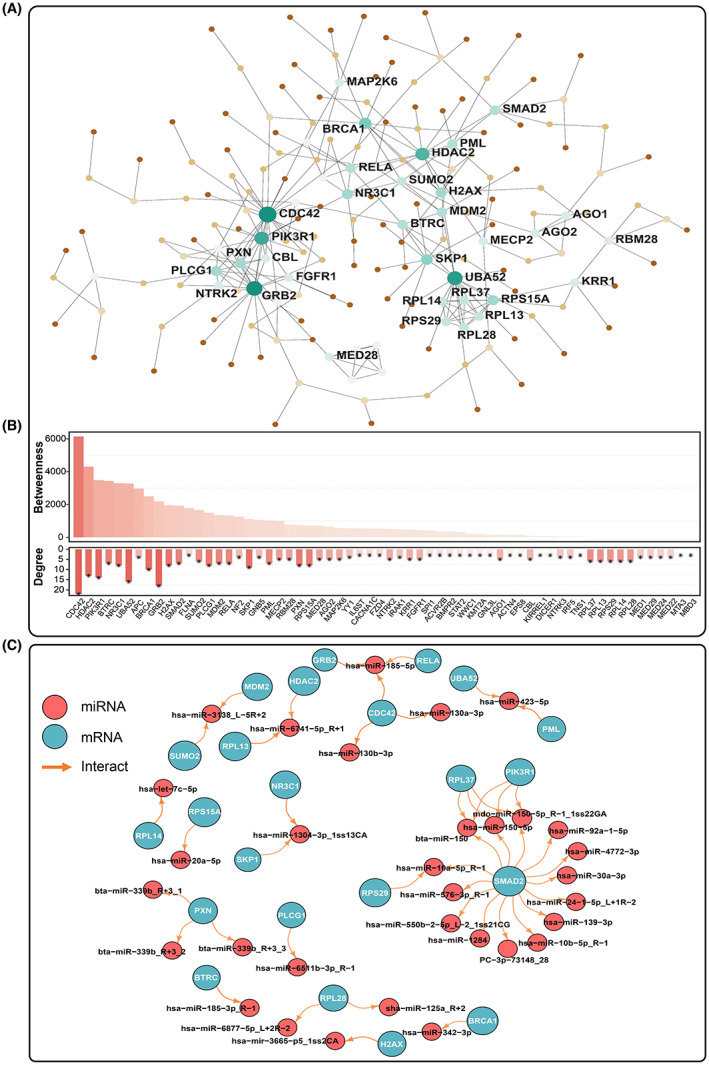
Gene correlation network analysis to identify hub genes in DCM accompanied with CHF. Every node represents one gene, and each edge represents the interaction between nodes. (A) Protein–protein interaction network of 156 target genes of DE‐miRNAs and 23 hub genes (degree >5) were annotated. Coloured and sized by degree (number of connections). (B) The parameters of target genes of DE‐miRNAs corresponding to PPI network. (C) miRNA‐mRNA interaction network. The red nodes represent miRNAs; the blue nodes represent hub genes.

The 1176 matched genes of DE‐miRNAs through TargetScan and miRanda were imported into the STRING database. Excluding the isolated targeted genes without interaction, there was a total of 156 target genes were mapped into the PPI network (confidence score cut‐off value 900), which comprised with 156 nodes and 263 edges. We also used Cytoscape software to visualize the interactions between the genes (Figure 5A). According to the CytoHubba plugin compute, 23 nodes with a degree >5 were confirmed as hub genes in the PPI network. These 23 hub genes are presented in Figure 5B and Table 2.

**TABLE 2:** Topology parameters of hub genes (degree >5) in the PPI network.

Gene	Degree	Betweenness	Gene	Degree	Betweenness
CDC42	22	6147.15	BTRC	7	3441.49
GRB2	18	2188.94	SMAD2	7	1926.59
UBA52	16	3280.12	MDM2	7	1362.84
PIK3R1	14	3485.67	RELA	7	1322.47
HDAC2	13	4306.73	PML	7	1032.14
BRCA1	10	2497.89	SUMO2	6	1662.36
SKP1	9	1113.25	RPL37	6	0
NR3C1	8	3290.88	RPL13	6	0
H2AX	8	1957.74	RPS29	6	0
PLCG1	8	1498.55	RPL14	6	0
PXN	8	749.75	RPL28	6	0
RPS15A	8	720.37			

The specific roles of DE‐miRNAs and their target hub genes were further investigated using the comprehensive biological annotation analysis (Figure S1). Based on WikiPathway annotation (https://www.wikipathways.org/), we found these DE‐miRNAs were mainly involved in the PDGF pathway (WP2526), leptin signalling (WP2034) and T‐cell receptor (TCR) signalling pathway (WP69). BioCarta database analysis (https://cgap.nci.nih.gov/Pathways/BioCarta_Pathways) showed DE‐miRNAs and their target hub genes played a major role in the VEGF, hypoxia and angiogenesis pathways. Furthermore, BioPlanet database annotation confirmed they were significantly associated with the activation of signalling mediated by EGFR, VEGF and VEGFR.

### 3.9 miRNA–mRNA interaction network analysis

Aiming to understand the molecular mechanisms of previously identified exosomal DE‐miRNAs in DCM accompanied with CHF, miRNet was used to recognize miRNAs targeting hub genes. As a result, a miRNA–mRNA regulatory network with 55 nodes (32 miRNAs and 23 hub genes) and 59 edges was constructed (Figure 5C).

Notably, we found that 9 DE‐miRNAs which contemporaneously targeted two or more hub genes, including hsa‐miR‐185‐5p, bta‐miR‐150, mdo‐miR‐150‐5p_R‐1_1ss22GA, hsa‐miR‐150‐5p, hsa‐miR‐423‐5p, hsa‐miR‐6741‐5p_R+1, hsa‐miR‐1304‐3p_1ss13CA, hsa‐miR‐10a‐5p_R‐1 and hsa‐miR‐3138_L‐5R+2. Additionally, it is also intriguing that the hub gene SMAD2 have the highest overlapping target number with DE‐miRNAs (PC‐3p‐73148_28, bta‐miR‐150, hsa‐miR‐10a‐5p_R‐1, hsa‐miR‐10b‐5p_R‐1, hsa‐miR‐1284, hsa‐miR‐139‐3p, hsa‐miR‐150‐5p, hsa‐miR‐24‐1‐5p_L+1R‐2, hsa‐miR‐30a‐3p, hsa‐miR‐4772‐3p, hsa‐miR‐550b‐2‐5p_L‐2_1ss21CG, hsa‐miR‐576‐3p_R‐1, mdo‐miR‐150‐5p_R‐1_1ss22GA).

### 3.10 Validation of hub miRNAs performance in an external cohort

The top 10 miRNAs that were significantly dysregulated between DCM and healthy individuals in silico were identified as hub miRNAs (hsa‐miR‐6511b‐3p, hsa‐miR‐6741‐5p, hsa‐miR‐1292‐5p, hsa‐miR‐6877‐5p, hsa‐miR‐423‐5p, hsa‐miR‐3138, hsa‐miR‐1304‐3p, hsa‐miR‐5010‐5p, hsa‐mir‐148a‐3p, hsa‐miR‐1306‐3p). We next validated the performance of top hub miRNAs in an external population (Exo from 10 patients and six healthy individuals) based on qRT‐PCR.

**FIGURE 6:**
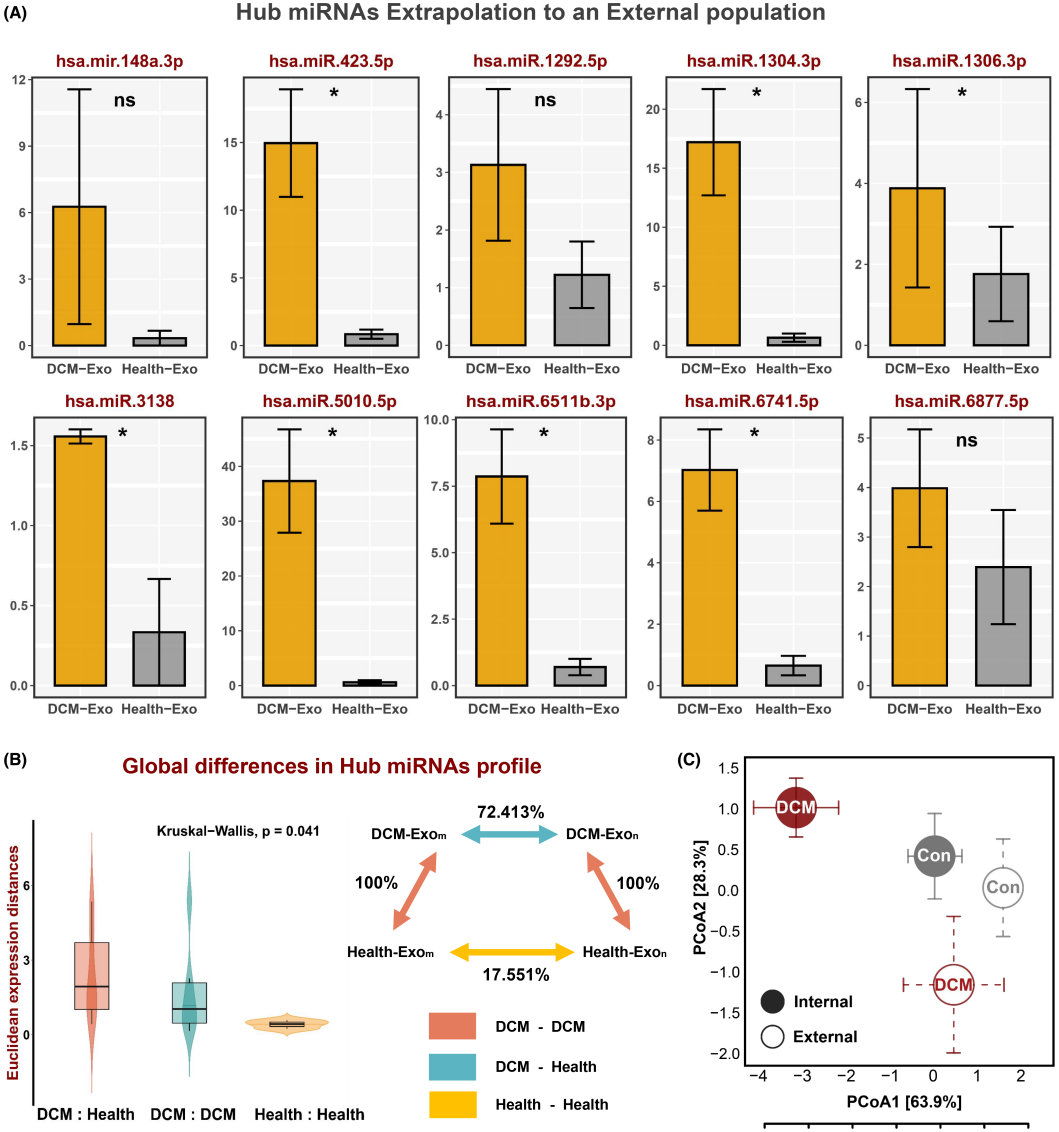
Validation of hub miRNAs performance in external cohort. (A) The distribution of 10 hub miRNAs expression level between DCM and healthy control groups in the external cohort based on qRT‐PCR. *p < 0.05 (Wilcoxon test) and ns represents no significance. (B) Global differences in 10 hub miRNAs expression between DCM and healthy control groups in the external cohort. The Euclidean expression distances were calculated between DCM and controls (red), different samples of DCM (green) and different samples of controls (yellow). The inset summarizes the average distances between pairs of samples as a percentage of the average distance between DCM and controls. (C) Principle coordinate analysis of Bray–Curtis dissimilarities obtained for the 10 hub miRNAs expression profiles in the internal (closed circles) and external (open circles) cohorts. The circles and error bars indicated the mean and standard errors of the mean.

Our analysis demonstrated a significant upregulation of hub miRNAs in the DCM samples, which further supported the results in silico (Figure 6A). We then applied one measure of divergence between a pair of expression profiles, the Euclidean distance to investigate the global shifts in the hub miRNA expression profile between and within DCM and healthy controls in the external cohort. Relative differences between the distributions were consistent for this metric of expression divergence. The expression distance between DCM and healthy controls or within the DCM samples was significantly larger than the distance within normal healthy controls (Figure 6B). Principal coordinates analysis (PCoA) of hub miRNA profile in the internal silico cohort showed that significant shifts separated DCM from healthy controls. Similar results were observed from our external independent cohort, where there was a significant difference in the hub miRNA profiles between them (Figure 6C).

## 4 DISCUSSION

In the present study, a specific miRNA signature of plasma exosomes was identified in DCM accompanied with CHF patients. We screened 92 DE‐miRNAs related to DCM accompanied with CHF using miRNA sequencing. Forty‐eight upregulated and 44 downregulated miRNAs were identified, and 75 targets of these DE‐miRNAs were confirmed. To predict the function of dysregulation miRNAs in DCM accompanied with CHF, GO and KEGG analyses were further performed. Some enriched pathways were enunciated. Particularly, oxytocin signalling pathway, circadian entrainment and hippo signalling pathway‐multiple species were listed as the top three.

In addition to its distinguished role played in lactation and parturition, oxytocin signalling pathway also involved in many other physiological functions and pathological processes, such as the maintenance of blood supply to the cortex, the regulation of the growth of the neocortex and the modulation of the autonomic nervous system via the vagal pathway to name a few. Additionally, it also demonstrated anti‐atherogenic, anti‐oxidant, anti‐inflammatory andanti‐dyslipidaemic effects.^35^ The reperfusion injury salvage kinase (RISK) signal pathway is an essential signal cascade of the cardioprotective mechanism after ischemic postconditioning. Previous studies have reported that the administration of oxytocin remarkedly reduced the infarct size, incidence of ventricular fibrillation, arrhythmia score, concomitantly increased coronary flow in rat heart. It exerts these cardioprotective effects through activating RISK signal pathway through the ERK1/2 and PI3K/Akt cascades.^36^ Similar with above‐mentioned studies, Plante et al. found that oxytocin treatment could prevented the cardiomyopathy in obese diabetic male db/db mice via partially improving fat and glucose metabolism and preventing systolic and diastolic dysfunction, cardiomyocyte hypertrophy, fibrosis as well as apoptosis.^37^ Furthermore, Alizadeh et al. demonstrated that oxytocin protects rat heart from ischemia/reperfusion injury through the pathway of mitochondrial ATP‐dependent potassium channel.^38^ Thus, it could be speculated that the DE‐miRNAs enriched in oxytocin signalling pathway may also possess cardioprotective effect in patients of DCM accompanied with CHF.

Circadian entrainment is endogenous oscillations, widely found in biological species, that possess the ability of entraining to the 24 h light–dark cycle. Studies have revealed that circadian dysregulation is closely associated with increased risk of cardiovascular diseases, including hypertension, atherosclerosis, stroke and myocardial infarction.^39^ The nuclear receptor Rev‐erbα/β, the key constituent of the circadian clock, emerges as a drug target for cardiovascular diseases. Rev‐erb agonists can attenuate pressure overload–resulted myocardial infarction, cardiac hypertrophy or myocardial ischemia/reperfusion in mice.^40^, ^41^, ^42^, ^43^ Rev‐erb antagonist aggrandizes the tolerance of myocardial ischemia/reperfusion ex vivo at the sleep‐to‐wake transition.^44^ Interestingly, Song et al. conducted a retrospective analysis of the clock gene expression in failing hearts from DCM patients who receive heart transplantation. The result of that study suggests that the cardiac molecular chronotype is relevant with the severity of cardiac dilation in DCM patients.^45^

Hippo signalling pathway is a functionally and evolutionarily conserved regulator of organ size and growth with essential roles in cell differentiation, proliferation and apoptosis. Transgenic and gene knockout mouse models have revealed that the Hippo signalling pathway is participated in cardiomyocyte proliferation, heart development, hypertrophy, apoptosis and cardiac regeneration.^46^, ^47^, ^48^, ^49^, ^50^, ^51^, ^52^ Dysregulation of the Hippo signalling pathway causes divers kinds of heart diseases, including cardiac hypertrophy, atherosclerosis, myocardial infarction and neointima formation. One study found that Hippo signalling activation evokes mitochondrial damage via suppressing mitochondrial genes in mice, which causally contributes to the development of DCM.^53^ Therefore, the Hippo pathway maybe represents an emerging therapeutic target against mitochondrial dysfunction in DCM.

Nine dysregulated exosomal miRNAs related to DCM with CHF were further screened out, among which hsa‐miR‐185‐5p, hsa‐miR‐423‐5p, hsa‐miR‐6741‐5p_R+1, hsa‐miR‐3138_L‐5R+2 and hsa‐miR‐1304‐3p_1ss13CA were upregulated, bta‐miR‐150, mdo‐miR‐150‐5p_R‐1_1ss22GA, hsa‐miR‐150‐5p and hsa‐miR‐10a‐5p_R‐1 were downregulated. Some of these dysregulated miRNAs have been reported. For example, consistent with our current study, miR‐423‐5p is increased in the plasma of patients of heart failure caused by DCM, and the expression level of miR‐423‐5p in the plasma is positively related to the level of N‐terminal pro‐brain natriuretic peptide (NT‐proBNP).^54^ Lin et al. have found that miR‐185‐5p can facilitate myocardial fibrosis by targeting apelin receptor.^55^ The hsa‐miR‐185‐5p has been shown to regulate cardiac fibrosis and remodelling in DCM through the TGF‐β signalling pathway.^56^ hsa‐miR‐423‐5p has been reported to be involved in the regulation of angiogenesis and cardiac fibrosis in DCM.^57^ hsa‐miR‐6741‐5p_R+1 is a novel miRNA that has not yet been extensively studied in DCM, but has been shown to be associated with cardiomyocyte hypertrophy in other cardiovascular diseases.^58^ hsa‐miR‐3138_L‐5R+2 has been found to regulate mitochondrial function in cardiac cells and protect against DCM‐related cardiomyocyte apoptosis.^59^ hsa‐miR‐1304‐3p_1ss13CA has been reported to regulate inflammation and oxidative stress in the context of DCM.^60^ bta‐miR‐150 and mdo‐miR‐150‐5p_R‐1_1ss22GA are both members of the miR‐150 family, and have been shown to be involved in the regulation of cardiac hypertrophy, fibrosis and inflammation.^61^, ^62^ hsa‐miR‐150‐5p has also been shown to regulate angiogenesis and cardiac remodelling in DCM, possibly through the PI3K/AKT signalling pathway.^63^ Finally, hsa‐miR‐10a‐5p_R‐1 has been found to regulate cardiac fibrosis in DCM through the TGF‐β/Smad3 signalling pathway.^64^

In summary, the identified 9 miRNAs have been found to play important roles in the pathogenesis of DCM through a variety of mechanisms, including regulation of fibrosis, hypertrophy, inflammation, oxidative stress, angiogenesis and mitochondrial function. Further studies are needed to elucidate the exact mechanisms by which these miRNAs are involved in DCM, which may contribute to the development of novel diagnostic and therapeutic strategies for this debilitating disease.

Several limitations are existed in this study. Firstly, the study sample was relatively small, and the patients who were included were strictly DCM with CHF subjects. Secondly, limited evidence indicates the circulating miRNAs secretion into the extracellular space were from the heart directly. Hence, additional experiments on human heart samples are valuable to further confirm these results and bioinformatic predictions. Moreover, the related mechanism about how differentially expressed miRNAs participate in the pathological process of DCM requires further exploration.

In conclusion, our presented study identified the differentially expressed exosomal miRNAs in DCM with CHF patients and revealed the underlying pathological changes of DCM with CHF, which may also provide novel diagnosis and therapeutic methods of DCM.

## AUTHOR CONTRIBUTIONS

**Li Zhang:** Conceptualization (equal); data curation (equal); formal analysis (equal); funding acquisition (equal); investigation (equal); methodology (equal); project administration (equal); resources (equal); software (equal); supervision (equal); validation (equal); visualization (equal); writing – original draft (equal); writing – review and editing (equal). **Ge Zhang:** Validation (equal); visualization (equal); writing – original draft (equal); writing – review and editing (equal). **Yongzheng Lu:** Validation (equal); visualization (equal); writing – original draft (equal); writing – review and editing (equal). **JIamin Gao:** Validation (equal); visualization (equal); writing – original draft (equal); writing – review and editing (equal). **Zhen Qin:** Validation (equal); visualization (equal); writing – original draft (equal); writing – review and editing (equal). **Shuai Xu:** Validation (equal); visualization (equal); writing – original draft (equal); writing – review and editing (equal). **Zeyu Wang:** Validation (equal); visualization (equal); writing – original draft (equal); writing – review and editing (equal). **Yanyan Xu:** Validation (equal); visualization (equal); writing – original draft (equal); writing – review and editing (equal). **Yu Yang:** Validation (equal); visualization (equal); writing – original draft (equal); writing – review and editing (equal). **Jinying Zhang:** Conceptualization (equal); data curation (equal); formal analysis (equal); funding acquisition (equal); investigation (equal); methodology (equal); project administration (equal); resources (equal); software (equal); supervision (equal); validation (equal); visualization (equal); writing – original draft (equal); writing – review and editing (equal). **Junnan Tang:** Conceptualization (lead); data curation (lead); formal analysis (lead); funding acquisition (lead); investigation (lead); methodology (lead); project administration (lead); resources (lead); software (lead); supervision (lead); validation (lead); visualization (lead); writing – original draft (lead); writing – review and editing (lead).

## FUNDING INFORMATION

This work was supported by the National Natural Science Foundation of China (No. 82222007, 82170281 and U2004203), the Henan Thousand Talents Program (No. ZYQR201912131), Excellent Youth Science Foundation of Henan Province (No. 202300410362) and Central Plains Youth Top Talent, Advanced funds (No. 2021‐CCA‐ACCESS‐125).

## CONFLICT OF INTEREST STATEMENT

The authors declare no conflicts of interest.

## Supporting information


Figure S1

Table S1

Table S2

Table S3

## Data Availability

Any additional information required to reanalyse the data reported in this paper is available from the corresponding author contact upon request.

## References

[1] Gillum RF . Idiopathic cardiomyopathy in the United States, 1970‐1982. Am Heart J. 1986;111(4):752‐755. 3513505 10.1016/0002-8703(86)90111-0

[2] Manolio TA , Baughman KL , Rodeheffer R , et al. Prevalence and etiology of idiopathic dilated cardiomyopathy (summary of a National Heart, Lung, and Blood Institute workshop). Am J Cardiol. 1992;69(17):1458‐1466. 1590237 10.1016/0002-9149(92)90901-a

[3] Stergiopoulos K , Lima FV . Peripartum cardiomyopathy‐diagnosis, management, and long term implications. Trends Cardiovasc Med. 2019;29(3):164‐173. doi:10.1016/j.tcm.2018.07.012 30111492

[4] Masarone D , Kaski JP , Pacileo G , et al. Epidemiology and clinical aspects of genetic cardiomyopathies. Heart Fail Clin. 2018;14(2):119‐128. doi:10.1016/j.hfc.2017.12.007 29525641

[5] Amoah AG , Kallen C . Aetiology of heart failure as seen from a National Cardiac Referral Centre in Africa. Cardiology. 2000;93(1–2):11‐18. 10894901 10.1159/000006996

[6] Hershberger RE , Hedges DJ , Morales A . Dilated cardiomyopathy: the complexity of a diverse genetic architecture. Nat Rev Cardiol. 2013;10(9):531‐547. doi:10.1038/nrcardio.2013.105 23900355

[7] Burke MA , Cook SA , Seidman JG , Seidman CE . Clinical and mechanistic insights into the genetics of cardiomyopathy. J Am Coll Cardiol. 2016;68(25):2871‐2886. doi:10.1016/j.jacc.2016.08.079 28007147 PMC5843375

[8] Japp AG , Gulati A , Cook SA , Cowie MR , Prasad SK . The diagnosis and evaluation of dilated cardiomyopathy. J Am Coll Cardiol. 2016;67(25):2996‐3010. doi:10.1016/j.jacc.2016.03.590 27339497

[9] Pugh TJ , Kelly MA , Gowrisankar S , et al. The landscape of genetic variation in dilated cardiomyopathy as surveyed by clinical DNA sequencing. Genet Med. 2014;16(8):601‐608. doi:10.1038/gim.2013.204 24503780

[10] Marian AJ , van Rooij E , Roberts R . Genetics and genomics of single‐gene cardiovascular diseases: common hereditary cardiomyopathies as prototypes of single‐gene disorders. J Am Coll Cardiol. 2016;68(25):2831‐2849. doi:10.1016/j.jacc.2016.09.968 28007145 PMC5189923

[11] Sweet M , Taylor MRG , Mestroni L . Diagnosis, prevalence, and screening of familial dilated cardiomyopathy. Expert Opin Orphan Drugs. 2015;3(8):869‐876. 27547593 10.1517/21678707.2015.1057498 PMC4988677

[12] Harakalova M , Kummeling G , Sammani A , et al. A systematic analysis of genetic dilated cardiomyopathy reveals numerous ubiquitously expressed and muscle‐specific genes. Eur J Heart Fail. 2015;17(5):484‐493. doi:10.1002/ejhf.255 25728127

[13] Cheng L , Sharples RA , Scicluna BJ , Hill AF . Exosomes provide a protective and enriched source of miRNA for biomarker profiling compared to intracellular and cell‐free blood. J Extracell Vesicles. 2014;3:3. doi:10.3402/jev.v3.23743 PMC3968297 24683445

[14] Turchinovich A , Weiz L , Langheinz A , Burwinkel B . Characterization of extracellular circulating microRNA. Nucleic Acids Res. 2011;39(16):7223‐7233. doi:10.1093/nar/gkr254 21609964 PMC3167594

[15] Bartel DP . Metazoan MicroRNAs. Cell. 2018;173(1):20‐51. doi:10.1016/j.cell.2018.03.006 29570994 PMC6091663

[16] Kalluri R , LeBleu VS . The biology, function, and biomedical applications of exosomes. Science. 2020;367(6478):eaau6977. doi:10.1126/science.aau6977 32029601 PMC7717626

[17] Ge Q , Zhou Y , Lu J , Bai Y , Xie X , Lu Z . miRNA in plasma exosome is stable under different storage conditions. Molecules. 2014;19(2):1568‐1575. doi:10.3390/molecules19021568 24473213 PMC6271968

[18] Li H , Ouyang Y , Sadovsky E , Parks WT , Chu T , Sadovsky Y . Unique microRNA signals in plasma exosomes from pregnancies complicated by preeclampsia. Hypertension. 2020;75(3):762‐771. doi:10.1161/HYPERTENSIONAHA.119.14081 31983308 PMC7076905

[19] Jiang X , Li J , Zhang B , et al. Differential expression profile of plasma exosomal microRNAs in women with polycystic ovary syndrome. Fertil Steril. 2021;115(3):782‐792. doi:10.1016/j.fertnstert.2020.08.019 33041053

[20] Richardson P , McKenna W , Bristow M , et al. Report of the 1995 World Health Organization/international society and Federation of Cardiology Task Force on the definition and classification of cardiomyopathies. Circulation. 1996;93(5):841‐842. 8598070 10.1161/01.cir.93.5.841

[21] Jessup M , Abraham WT , Casey DE , et al. 2009 focused update: ACCF/AHA guidelines for the diagnosis and Management of Heart Failure in adults: a report of the American College of Cardiology Foundation/American Heart Association task force on practice guidelines: developed in collaboration with the International Society for Heart and Lung Transplantation. Circulation. 2009;119(14):1977‐2016. doi:10.1161/CIRCULATIONAHA.109.192064 19324967

[22] Cer RZ , Herrera‐Galeano JE , Anderson JJ , Bishop‐Lilly KA , Mokashi VP . miRNA temporal analyzer (mirnaTA): a bioinformatics tool for identifying differentially expressed microRNAs in temporal studies using normal quantile transformation. Gigascience. 2014;3:20. doi:10.1186/2047-217X-3-20 25379175 PMC4212236

[23] Zhang G , Cui X , Zhang L , et al. Uncovering the genetic links of SARS‐CoV‐2 infections on heart failure co‐morbidity by a systems biology approach. ESC Heart Fail. 2022;9(5):2937‐2954. doi:10.1002/ehf2.14003 35727093 PMC9349450

[24] Zhang G , Liu Z , Deng J , et al. Smooth muscle cell fate decisions decipher a high‐resolution heterogeneity within atherosclerosis molecular subtypes. J Transl Med. 2022;20(1):568. doi:10.1186/s12967-022-03795-9 36474294 PMC9724432

[25] Kore RA , Henson JC , Hamzah RN , et al. Molecular events in MSC exosome mediated cytoprotection in cardiomyocytes. Sci Rep. 2019;9(1):19276. doi:10.1038/s41598-019-55694-7 31848380 PMC6917778

[26] Betel D , Koppal A , Agius P , Sander C , Leslie C . Comprehensive modeling of microRNA targets predicts functional non‐conserved and non‐canonical sites. Genome Biol. 2010;11(8):R90. doi:10.1186/gb-2010-11-8-r90 20799968 PMC2945792

[27] Li D , Zhang G , Wang Z , et al. Idebenone attenuates ferroptosis by inhibiting excessive autophagy via the ROS‐AMPK‐mTOR pathway to preserve cardiac function after myocardial infarction. Eur J Pharmacol. 2023;943:175569. doi:10.1016/j.ejphar.2023.175569 36740037

[28] von Mering C , Huynen M , Jaeggi D , Schmidt S , Bork P , Snel B . STRING: a database of predicted functional associations between proteins. Nucleic Acids Res. 2003;31(1):258‐261. 12519996 10.1093/nar/gkg034 PMC165481

[29] Chang L , Zhou G , Soufan O , Xia J . miRNet 2.0: network‐based visual analytics for miRNA functional analysis and systems biology. Nucleic Acids Res. 2020;48(W1):W244‐W251. doi:10.1093/nar/gkaa467 32484539 PMC7319552

[30] Hu J , Locasale JW , Bielas JH , et al. Heterogeneity of tumor‐induced gene expression changes in the human metabolic network. Nat Biotechnol. 2013;31(6):522‐529. doi:10.1038/nbt.2530 23604282 PMC3681899

[31] Zhang G , Gong S , Pang L , Hou L , He W . Efficacy and safety of Apatinib treatment for advanced cholangiocarcinoma after failed gemcitabine‐based chemotherapy: an open‐label phase II prospective study. Front Oncol. 2021;11:659217. doi:10.3389/fonc.2021.659217 34012920 PMC8126718

[32] De Stefano ME , Ferretti V , Mozzetta C . Synaptic alterations as a neurodevelopmental trait of Duchenne muscular dystrophy. Neurobiol Dis. 2022;168:105718. doi:10.1016/j.nbd.2022.105718 35390481

[33] Backes C , Rühle F , Stoll M , et al. Systematic permutation testing in GWAS pathway analyses: identification of genetic networks in dilated cardiomyopathy and ulcerative colitis. BMC Genomics. 2014;15:622. doi:10.1186/1471-2164-15-622 25052024 PMC4223581

[34] Sabharwal R , Weiss RM , Zimmerman K , Domenig O , Cicha MZ , Chapleau MW . Angiotensin‐dependent autonomic dysregulation precedes dilated cardiomyopathy in a mouse model of muscular dystrophy. Exp Physiol. 2015;100(7):776‐795. doi:10.1113/EP085066 25921929 PMC4505616

[35] Iovino M , Messana T , Tortora A , et al. Oxytocin signaling pathway: from cell biology to clinical implications. Endocr Metab Immune Disord Drug Targets. 2021;21(1):91‐110. doi:10.2174/1871530320666200520093730 32433011

[36] Polshekan M , Jamialahmadi K , Khori V , et al. RISK pathway is involved in oxytocin postconditioning in isolated rat heart. Peptides. 2016;86:55‐62. doi:10.1016/j.peptides.2016.10.001 27717750

[37] Plante E , Menaouar A , Danalache BA , et al. Oxytocin treatment prevents the cardiomyopathy observed in obese diabetic male db/db mice. Endocrinology. 2015;156(4):1416‐1428. doi:10.1210/en.2014-1718 25562615

[38] Alizadeh AM , Faghihi M , Sadeghipour HR , et al. Oxytocin protects rat heart against ischemia‐reperfusion injury via pathway involving mitochondrial ATP‐dependent potassium channel. Peptides. 2010;31(7):1341‐1345. doi:10.1016/j.peptides.2010.04.012 20417240

[39] Martino TA , Young ME . Influence of the cardiomyocyte circadian clock on cardiac physiology and pathophysiology. J Biol Rhythm. 2015;30(3):183‐205. doi:10.1177/0748730415575246 25800587

[40] Zhang L , Zhang R , Tien C‐L , et al. REV‐ERBα ameliorates heart failure through transcription repression. JCI Insight. 2017;2(17):e95177. doi:10.1172/jci.insight.95177 28878135 PMC5621902

[41] Stujanna EN , Murakoshi N , Tajiri K , et al. Rev‐erb agonist improves adverse cardiac remodeling and survival in myocardial infarction through an anti‐inflammatory mechanism. PLoS One. 2017;12(12):e0189330. doi:10.1371/journal.pone.0189330 29232411 PMC5726719

[42] Reitz CJ , Alibhai FJ , Khatua TN , et al. SR9009 administered for one day after myocardial ischemia‐reperfusion prevents heart failure in mice by targeting the cardiac inflammasome. Commun Biol. 2019;2:353. doi:10.1038/s42003-019-0595-z 31602405 PMC6776554

[43] Alibhai FJ , LaMarre J , Reitz CJ , et al. Disrupting the key circadian regulator CLOCK leads to age‐dependent cardiovascular disease. J Mol Cell Cardiol. 2017;105:24‐37. doi:10.1016/j.yjmcc.2017.01.008 28223222

[44] Montaigne D , Marechal X , Modine T , et al. Daytime variation of perioperative myocardial injury in cardiac surgery and its prevention by rev‐Erbα antagonism: a single‐Centre propensity‐matched cohort study and a randomised study. Lancet. 2018;391(10115):59‐69. doi:10.1016/S0140-6736(17)32132-3 29107324

[45] Song S , Tien C‐L , Cui H , et al. Myocardial rev‐erb‐mediated diurnal metabolic rhythm and obesity paradox. Circulation. 2022;145(6):448‐464. doi:10.1161/CIRCULATIONAHA.121.056076 35034472 PMC8812427

[46] McPherson JP , Tamblyn L , Elia A , et al. Lats2/Kpm is required for embryonic development, proliferation control and genomic integrity. EMBO J. 2004;23(18):3677‐3688. 15343267 10.1038/sj.emboj.7600371 PMC517611

[47] Heallen T , Zhang M , Wang J , et al. Hippo pathway inhibits Wnt signaling to restrain cardiomyocyte proliferation and heart size. Science. 2011;332(6028):458‐461. doi:10.1126/science.1199010 21512031 PMC3133743

[48] Song H , Mak KK , Topol L , et al. Mammalian Mst1 and Mst2 kinases play essential roles in organ size control and tumor suppression. Proc Natl Acad Sci U S A. 2010;107(4):1431‐1436. doi:10.1073/pnas.0911409107 20080598 PMC2824397

[49] Matsui Y , Nakano N , Shao D , et al. Lats2 is a negative regulator of myocyte size in the heart. Circ Res. 2008;103(11):1309‐1318. doi:10.1161/CIRCRESAHA.108.180042 18927464 PMC2775813

[50] Xin M , Kim Y , Sutherland LB , et al. Hippo pathway effector yap promotes cardiac regeneration. Proc Natl Acad Sci U S A. 2013;110(34):13839‐13844. doi:10.1073/pnas.1313192110 23918388 PMC3752208

[51] Xin M , Kim Y , Sutherland LB , et al. Regulation of insulin‐like growth factor signaling by yap governs cardiomyocyte proliferation and embryonic heart size. Sci Signal. 2011;4(196):ra70. doi:10.1126/scisignal.2002278 22028467 PMC3440872

[52] von Gise A , Lin Z , Schlegelmilch K , et al. YAP1, the nuclear target of hippo signaling, stimulates heart growth through cardiomyocyte proliferation but not hypertrophy. Proc Natl Acad Sci U S A. 2012;109(7):2394‐2399. doi:10.1073/pnas.1116136109 22308401 PMC3289361

[53] Wu W , Ziemann M , Huynh K , et al. Activation of hippo signaling pathway mediates mitochondria dysfunction and dilated cardiomyopathy in mice. Theranostics. 2021;11(18):8993‐9008. doi:10.7150/thno.62302 34522223 PMC8419046

[54] Fan K‐L , Zhang H‐F , Shen J , Zhang Q , Li X‐L . Circulating microRNAs levels in Chinese heart failure patients caused by dilated cardiomyopathy. Indian Heart J. 2013;65(1):12‐16. doi:10.1016/j.ihj.2012.12.022 23438607 PMC3860780

[55] Lin R , Rahtu‐Korpela L , Szabo Z , et al. MiR‐185‐5p regulates the development of myocardial fibrosis. J Mol Cell Cardiol. 2022;165:130‐140. doi:10.1016/j.yjmcc.2021.12.011 34973276

[56] Saincher RR , Pentapati KC , Gadicherla S . Effect of audio‐visual treatment information on hemodynamic parameters during the Transalveolar extraction of mandibular third molars: a randomized clinical trial. J Int Soc Prev Community Dent. 2019;9(1):21‐26. doi:10.4103/jispcd.JISPCD_366_18 30923689 PMC6402245

[57] Bursa F , Yellowlees A , Bishop A , Beckett A , Hallis B , Matheson M . Estimation of ELISA results using a parallel curve analysis. J Immunol Methods. 2020;486:112836. doi:10.1016/j.jim.2020.112836 32827492

[58] Krynicki CR , Upthegrove R , Deakin JFW , Barnes TRE . The relationship between negative symptoms and depression in schizophrenia: a systematic review. Acta Psychiatr Scand. 2018;137(5):380‐390. doi:10.1111/acps.12873 29532909

[59] Kohanski MA , Palmer JN , Adappa ND . Indications and endonasal treatment of petrous apex cholesterol granulomas. Curr Opin Otolaryngol Head Neck Surg. 2019;27(1):54‐58. doi:10.1097/MOO.0000000000000511 30507687

[60] Muranishi Y , Tanaka N , Kono T , Miyahara R . A case of intrapulmonary lymphangioma surrounded by pulmonary hilar structures. Respir Investig. 2020;58(6):506‐509. doi:10.1016/j.resinv.2020.04.006 32576446

[61] Zahid M , Rinas U . Guidelines for small‐scale production and purification of hepatitis B surface antigen virus‐like particles from recombinant Pichia pastoris. Methods Mol Biol. 2019;1923:309‐322. doi:10.1007/978-1-4939-9024-5_14 30737747

[62] Sigterman TA , Bolt LJJ , Snoeijs MG , et al. Radiation exposure during percutaneous transluminal angioplasty for symptomatic peripheral arterial disease. Ann Vasc Surg. 2016;33:167‐172. doi:10.1016/j.avsg.2015.11.019 26902938

[63] Dover EN , Beck R , Huang MC , et al. Arsenite and methylarsonite inhibit mitochondrial metabolism and glucose‐stimulated insulin secretion in INS‐1 832/13 β cells. Arch Toxicol. 2018;92(2):693‐704. doi:10.1007/s00204-017-2074-y 28956099 PMC6640649

[64] Linardi D , Walpoth B , Mani R , et al. Slow versus fast rewarming after hypothermic circulatory arrest: effects on neuroinflammation and cerebral oedema. Eur J Cardiothorac Surg. 2020;58(4):792‐800. doi:10.1093/ejcts/ezaa143 32408343

