# Supplementary material for: Differential expression profiles of plasma exosomal microRNAs in dilated cardiomyopathy with chronic heart failure
Source: J Cell Mol Med. 2023 May 27;27(14):1988–2003. doi: 10.1111/jcmm.17789 (PMC10339102; doi:10.1111/jcmm.17789)
Supplement: Supplementary file 1 — Figure S1 [file JCMM-27-1988-s002.pdf]

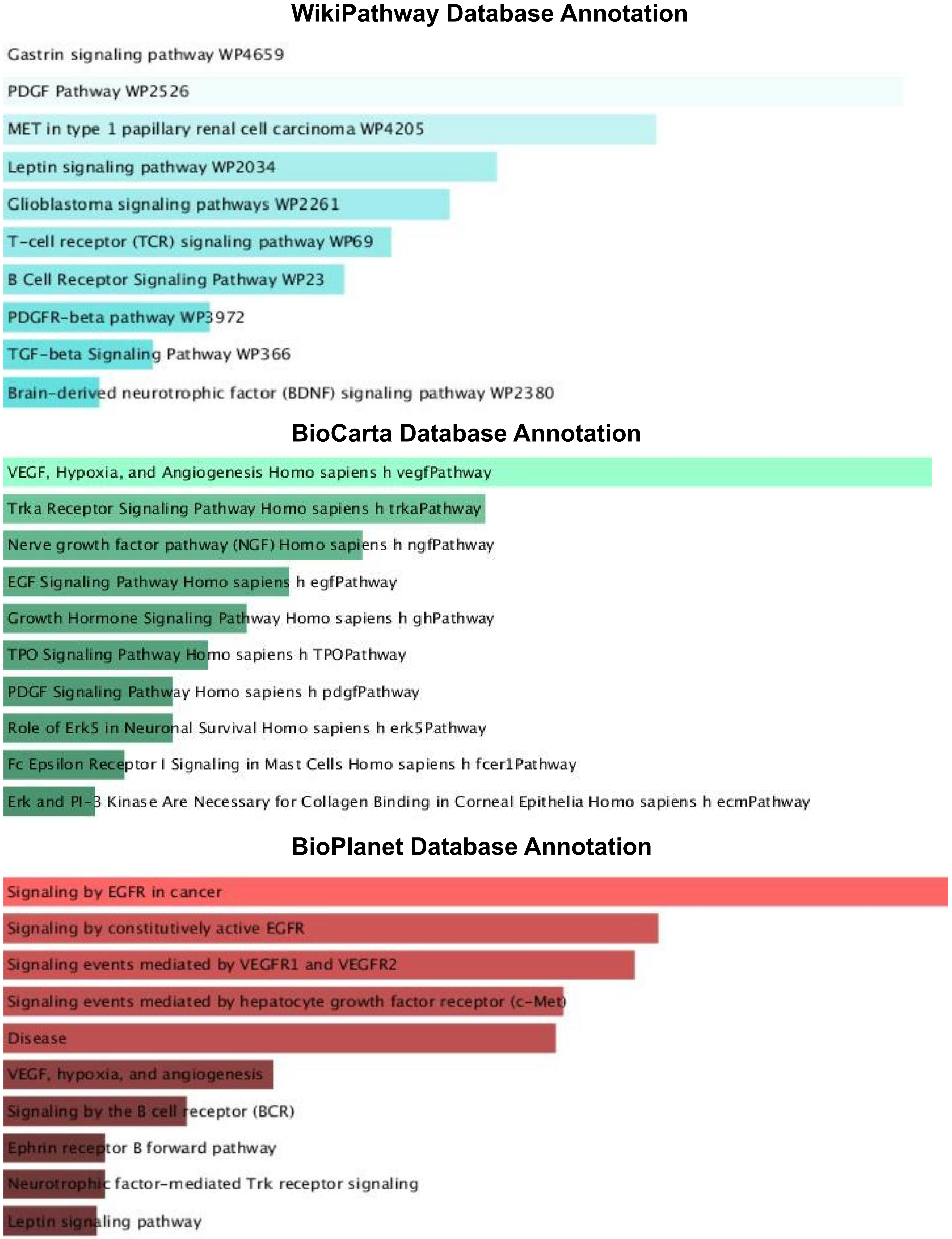
WikiPathway Database Annotation
Gastrin signaling pathway WP4659
PDGF Pathway WP2526
MET in type 1 papillary renal cell carcinoma WP4205
Leptin signaling pathway WP2034
Glioblastoma signaling pathways WP2261
T-cell receptor (TCR) signaling pathway WP69
B Cell Receptor Signaling Pathway WP23
PDGFR-beta pathway WP3972
TGF-beta Signaling Pathway WP366
Brain-derived neurotrophic factor (BDNF) signaling pathway WP2380
BioCarta Database Annotation
VEGF, Hypoxia, and Angiogenesis Homo sapiens h vegfPathway
Trka Receptor Signaling Pathway Homo sapiens h trkaPathway
Nerve growth factor pathway (NGF) Homo sapiens h ngfPathway
EGF Signaling Pathway Homo sapiens h egfPathway
Growth Hormone Signaling Pathway Homo sapiens h ghPathway
TPO Signaling Pathway Homo sapiens h TPOPathway
PDGF Signaling Pathway Homo sapiens h pdgfPathway
Role of Erk5 in Neuronal Survival Homo sapiens h erk5Pathway
Fc Epsilon Receptor I Signaling in Mast Cells Homo sapiens h fcer1Pathway
Erk and PI-3 Kinase Are Necessary for Collagen Binding in Corneal Epithelia Homo sapiens h ecmPathway
BioPlanet Database Annotation
Signaling by EGFR in cancer
Signaling by constitutively active EGFR
Signaling events mediated by VEGFR1 and VEGFR2
Signaling events mediated by hepatocyte growth factor receptor (c-Met)
Disease
VEGF, hypoxia, and angiogenesis
Signaling by the B cell receptor (BCR)
Ephrin receptor B forward pathway
Neurotrophic factor-mediated Trk receptor signaling
Leptin signaling pathway
